# Hybrid Imaging Agents for Pretargeting Applications Based on Fusarinine C—Proof of Concept

**DOI:** 10.3390/molecules25092123

**Published:** 2020-05-01

**Authors:** Dominik Summer, Milos Petrik, Sonja Mayr, Martin Hermann, Piriya Kaeopookum, Joachim Pfister, Maximilian Klingler, Christine Rangger, Hubertus Haas, Clemens Decristoforo

**Affiliations:** 1 Department of Nuclear Medicine, Medical University Innsbruck, A-6020 Innsbruck, Austria; summer.dominik@gmail.com (D.S.); sonja-mayr@hotmail.com (S.M.); gamsuk@hotmail.com (P.K.); joachim.pfister@i-med.ac.at (J.P.); Maximilian.klingler@i-med.ac.at (M.K.); Christine.Rangger@i-med.ac.at (C.R.); 2 Institute of Molecular and Translational Medicine, Faculty of Medicine and Dentistry, Palacky University Olomouc, 772-00 Olomouc, Czech Republic; MilosPetrik@upol.cz; 3 Department of Anaesthesia and Intensive Care, Medical University Innsbruck, A-6020 Innsbruck, Austria; Martin.Hermann@i-med.ac.at; 4 Institute of Molecular Biology, Medical University Innsbruck, A-6020 Innsbruck, Austria; Hubertus.Haas@i-med.ac.at

**Keywords:** fusarinine C, click chemistry, fluorescence, optical imaging, PET, gallium-68

## Abstract

Hybrid imaging combining the beneficial properties of radioactivity and optical imaging within one imaging probe has gained increasing interest in radiopharmaceutical research. In this study, we modified the macrocyclic gallium-68 chelator fusarinine C (FSC) by conjugating a fluorescent moiety and tetrazine (Tz) moieties. The resulting hybrid imaging agents were used for pretargeting applications utilizing click reactions with a *trans*-cyclooctene (TCO) tagged targeting vector for a proof of principle both in vitro and in vivo. Starting from FSC, the fluorophores Sulfocyanine-5, Sulfocyanine-7, or IRDye800CW were conjugated, followed by introduction of one or two Tz motifs, resulting in mono and dimeric Tz conjugates. Evaluation included fluorescence microscopy, binding studies, logD, protein binding, in vivo biodistribution, µPET (micro-positron emission tomography), and optical imaging (OI) studies. ^68^Ga-labeled conjugates showed suitable hydrophilicity, high stability, and specific targeting properties towards Rituximab-TCO pre-treated CD20 expressing Raji cells. Biodistribution studies showed fast clearance and low accumulation in non-targeted organs for both SulfoCy5- and IRDye800CW-conjugates. In an alendronate-TCO based bone targeting model the dimeric IRDye800CW-conjugate resulted in specific targeting using PET and OI, superior to the monomer. This proof of concept study showed that the preparation of FSC-Tz hybrid imaging agents for pretargeting applications is feasible, making such compounds suitable for hybrid imaging applications.

## 1. Introduction

**Scheme 1:**
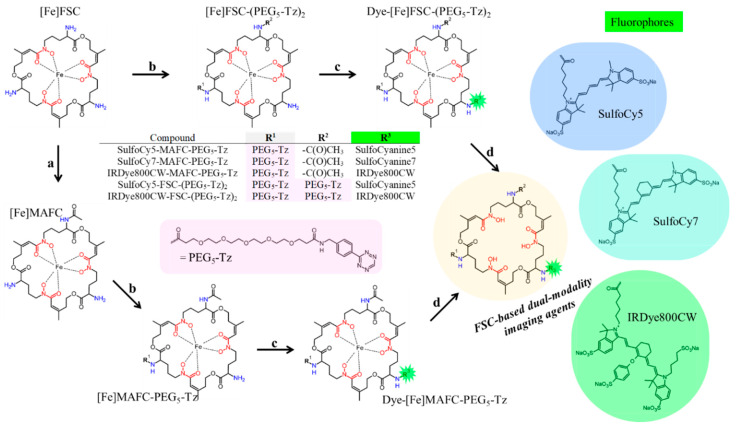
Route of synthesis for fusarinine C (FSC)-based clickable dual-modality imaging agents (**a**: MetOH/Ac_2_O; **b**: PEG_5_-Tz-NHS, DMF/DIPEA; **c**: fluorophore, DMF/DIPEA and *O*-(7-Azabenzotriazol-1-yl)-*N*,*N*,*N*′,*N*′-tetramethyluronium-hexafluorphosphate (HATU); **d**: EDTA).

Various imaging modalities have evolved as valuable tools for molecular imaging of human diseases. When used as a stand-alone technique, every method shows benefits and limitations [1] so combining different modalities, namely dual-modality- (DMI) or hybrid imaging (HI), is a field with increasing attraction. Optical imaging (OI), for example, is characterized by excellent sensitivity to acquire morphological information but poor tissue penetration limits its applicability for non-invasive imaging purposes. In contrast, single photon emission- (SPECT) and positron emission tomography (PET) show excellent tissue penetration and allow to obtain functional information with high sensitivity. However, due to the lack of morphological information unsurprisingly computed tomography (CT) or magnetic resonance (MR) are used to add morphological details. Although hybrid imaging systems like PET–CT/MRI and SPECT–CT are well established in clinical routine for high resolution imaging their value for real-time intra-operative guided surgery is limited. Despite recent advancements regarding Cerenkov luminescence imaging (CLI) [2] and the development of directional gamma probes (DGP) [3] for radio-guided surgery, optical imaging, particularly near-infrared fluorescence (NIRF) enables the delineation of tumor margins with high accuracy, thus bearing an enormous potential for image-guided surgery [4,5,6,7]. Developing targeted imaging probes to combine the complementary nature of PET and NIRF has gained increasing interest and various studies have shown promising results [8,9,10,11,12,13]. However, most of these studies rely on the use of small peptides as targeting structures being characterized by fast blood clearance and short biological half-life, thus exhibiting fast pharmacokinetics. In contrast, monoclonal antibodies (mAbs) are characterized by their prolonged circulation time exhibiting slow pharmacokinetics but their high affinity and excellent selectivity towards molecular targets bear great potential as imaging agents. The inverse electron-demand Diels-Alder (IEDDA) [14] reaction between 1,2,4,5-tetrazine (Tz) and trans-cyclooct-2-en (TCO) has been established successfully as a highly promising pretargeting strategy, where the TCO-modified mAb is administered prior to the injection of the Tz-bearing radioactive payload to form the radioimmunoconjugate in vivo with exquisite selectivity and tremendously rapid reaction kinetics [15]. With this the applicability of mAbs as targeting vectors has reached a new dimension. Zeglis and co-workers went one step further and have recently shown the suitability of mAbs as targeting vectors for PET/OI hybrid-imaging either using direct labelling [16] or following the IEDDA approach [17]. However, in both studies the fluorescent dye was directly attached to the mAb. In contrast, we herein report on the design of dual-modality imaging agents for pretargeting applications by chelator scaffolding i.e., the fluorescent residue was conjugated to the chelator. Therefore, we utilized the macrocyclic chelator fusarinine C (FSC), which has been already proven to be a suitable scaffold for the development of targeted hybrid imaging agents [18] and allows straight forward radiolabelling with Gallium-68 for PET applications without further modifications, providing advantages over, e.g., F-18 labelling. FSC provides three amine functionalities at the chelating backbone for site specific modification and despite the optical signalling residue we conjugated one or two Tz-motifs for potentially improved IEDDA-based pretargeting as presented in Scheme 1. We used Cyanine based fluorescent dyes as they provide a choice of wavelengths both for microscopy (Cy5) and near-infrared for OI (Cy7, IRDye800CW). A Polyethleneglycol-5 (PEG_5_)-Tetrazine was chosen for conjugation to FSC, which has provided suitable properties in a previous approach [19].

## 2. Results

### 2.1. Synthesis

The FSC-based Tz-bearing hybrid imaging agents were accessible by a straightforward three–four-step synthesis starting from the macrocyclic chelator [Fe]FSC in a similar approach as described in [18,19]. The monomeric conjugates were prepared by initial coupling of the Tetrazine-PEG_5_-NHS ester to the mono acetyl protected form of FSC, [Fe]MAFC, the dimeric conjugates by starting from [Fe]FSC. Using equal molar amounts the predominant products were [Fe]FSC-(Tetrazine-PEG_5_)_2_ or [Fe]MAFC-Tetrazine-PEG_5_, which were isolated by high-performance liquid chromatography (HPLC), whereby for [Fe]FSC yields were lower due to formation of [Fe]FSC-(Tetrazine-PEG_5_). The resulting conjugates were straight forward coupled with the respective Dyes by *O*-(7-Azabenzotriazol-1-yl)-*N*,*N*,*N′*,*N*′-tetramethyluronium-hexafluorphosphate (HATU) activation and finally iron was removed. The conjugates could be obtained in acceptable yield i.e., 30–60% for monomeric and 40–60% for dimeric FSC-based Tz hybrid imaging agents in sufficient purity (>90%) determined by analytical reversed phase (RP)-HPLC using UV absorption at λ = 220 nm.

### 2.2. Radiolabeling

**Figure 1:**
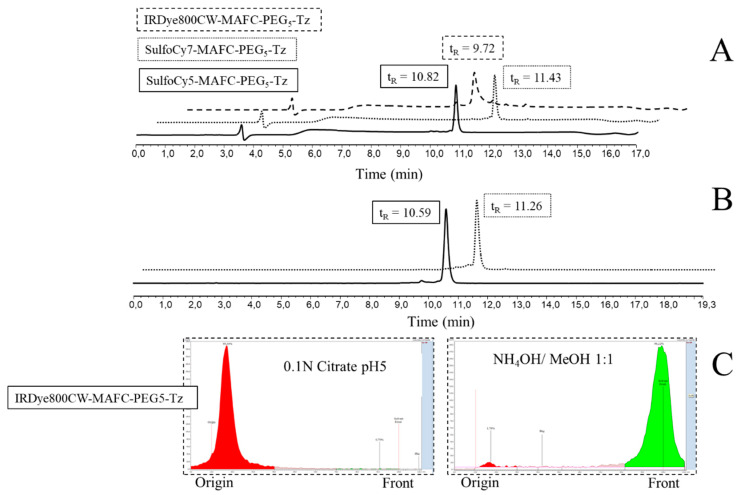
Representative chromatograms of monomeric FSC-based dual-modality imaging (DMI) agents. (**A**) shows UV-detection at λ = 220 nm, (**B**) represents radio detection of [^68^Ga]Ga-SulfoCy5-MAFC-PEG_5_-Tz and [^68^Ga]Ga-SulfoCy7-MAFC-PEG_5_-Tz, whereas (**C**) shows radio-ITLC (instant thin layer chromatography) analysis of [^68^Ga]Ga-IRDye800CW-MAFC-PEG_5_-Tz using citrate (left) and ammonium acetate/ethanol (right) as mobile phase.

Radiolabeling with the ^68^Ge/^68^Ga-generator derived radiometal gallium-68 was conducted at ambient temperature within five minutes and radiochemical yields greater than 98% determined by radio-RP-HPLC and radio-ITLC (instant thin layer chromatography) could be achieved—as exemplarily shown in Figure 1.

### 2.3. In Vitro Characterization

The results of the distribution coefficient logD and protein binding studies are presented in Table 1. All ^68^Ga-labeled conjugates showed reasonable hydrophilicity with values ranging from –1.92 for the most lipophilic (SulfoCy7-MAFC-PEG_5_-Tz) to –2.85 for the most hydrophilic conjugate (SulfoCy5-MAFC-PEG_5_-Tz). Interestingly, by replacing the acetyl residue by a second PEG_5_-Tz moiety the hydrophilicity was altered for the SulfoCy5-conjugate while for the corresponding IRDye800CW-conjugate it remained the same. The protein binding of the ^68^Ga-labeled FSC-based hybrid imaging agents was consistent over time and ranged from intermediate (30–50%) to high (50–70%). Introducing a SulfoCy5- or -Cy7 residue to the MAFC-Tz scaffold showed a comparable protein binding while the conjugation of the IRDye800CW led to significantly increased values. Interestingly, the replacement of the acetyl moiety by a PEG_5_-Tz residue did not increase the protein binding compared to the monomeric Tz conjugates. However, this is consistent with the results of a previous study where increasing the number of PEG_5_-Tz by chelator scaffolding did not increase the protein binding [19].

**Figure 2:**
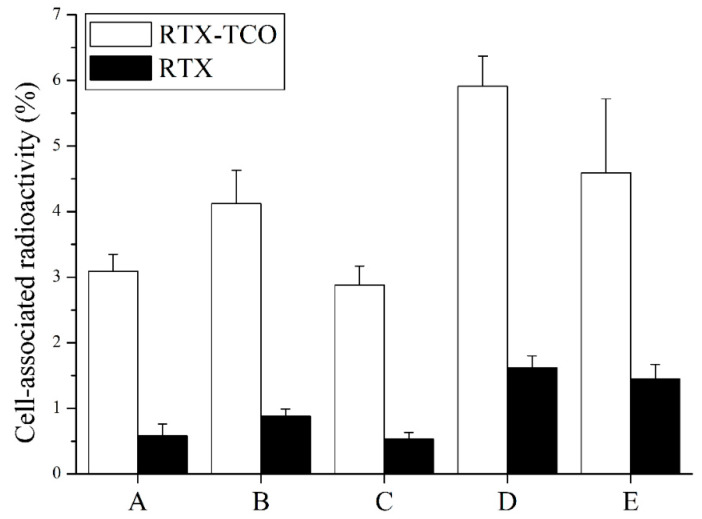
Cell binding studies of [^68^Ga]Ga-SulfoCy5-MAFC-PEG_5_-Tz (A), [^68^Ga]Ga-SulfoCy7-MAFC-PEG_5_-Tz (B), [^68^Ga]Ga-IRDye800CW-MAFC-PEG_5_-Tz (C), [^68^Ga]Ga-SulfoCy5-FSC-(PEG_5_-Tz)_2_ (D), and [^68^Ga]Ga-IRDye800CW-FSC-(PEG_5_-Tz)_2_ (E) on CD20-expressing Raji cells pre-treated with *trans*-cyclooctene (TCO) modified rituximab (white bars) and non-modified antibody (black bars).

The results of cell binding studies of the ^68^Ga-labeled FSC-based hybrid imaging agents on CD20-expressing Raji cells pre-treated with Rituximab(RTX)–TCO-modified or non-modified RTX are summarized in Figure 2. All conjugates showed highly specific targeting properties with ratios of specifically to non-specifically bound radioligand ranging from 3 to 5. The binding of the ^68^Ga-labeled monomeric DMI agents was 3.09 ± 0.58% for [^68^Ga]Ga-SulfoCy5-MAFC-PEG_5_-Tz, 4.12 ± 0.88% for [^68^Ga]Ga-SulfoCy7-MAFC-PEG_5_-Tz and 2.88 ± 0.53% for [^68^Ga]Ga-IRDye800CW-MAFC-PEG_5_-Tz which was comparable to the non-fluorescent conjugate [^68^Ga]Ga-DAFC-PEG_5_-Tz (4.01 ± 0.36%) [19]. The binding of the dimeric DMI agents radiolabeled with gallium-68 was significantly higher (*p* < 0.005) in comparison to their monomeric counterparts and resulted to be 5.91 ± 1.62% for [^68^Ga]Ga-SulfoCy5-FSC-(PEG_5_-Tz)_2_ and 4.59 ± 1.45% for [^68^Ga]Ga-IRDye800CW-FSC-(PEG_5_-Tz)_2_ but was significantly lower (*p* < 0.0005) compared to the ^68^Ga-labeled non-fluorescent conjugate [^68^Ga]Ga-MAFC-(PEG_5_-Tz)_2_ (7.35 ± 0.50%) [19]. Furthermore, the unspecific binding was significantly increased (*p* < 0.005) also when comparing ^68^Ga-labeled mono- and dimeric FSC-based DMI agents.

**Figure 3:**
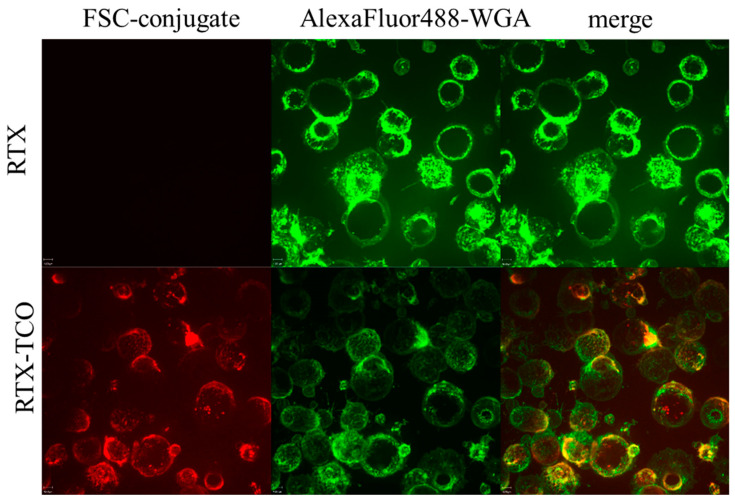
Fluorescence microscopy of CD20-expressing Raji cells pre-treated with RTX (upper lane) and with RTX–TCO (lower lane, negative control) prior to incubation with SulfoCy5-MAFC-PEG_5_-Tz. Each lane shows the fluorescence signal of the FSC-based conjugate (red), optical signal from AlexaFlour488-WGA labeled cells (green) and both images merged (from left to right).

Fluorescence microscopy of SulfoCy5-conjugates is presented in Figure 3 and clearly shows the high target specificity as the RTX–TCO pre-treated Raji cells revealed SulfoCy5-specific signaling whereas the negative control, lacking the TCO functionality, did not.

### 2.4. Biodistribution Studies

**Figure 4:**
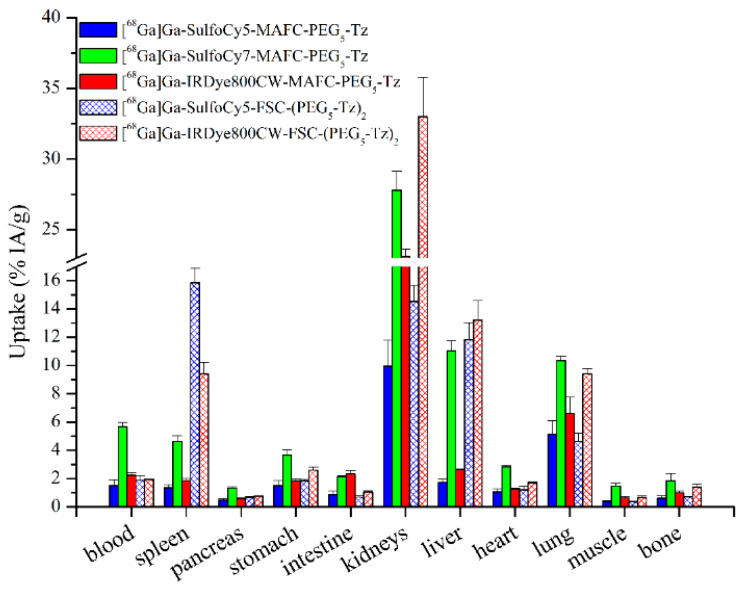
Ex vivo biodistribution profile studies of ^68^Ga-labeled FSC-based Tz-bearing dual-modality imaging agents in healthy BALB/c mice 1 h after administration.

The ex vivo biodistribution profile of the ^68^Ga-labeled FSC-based hybrid imaging agents in healthy BALB/C mice 1 h post injection (p.i.) is summarized in Figure 4. Except from the SulfoCy7-conjugate all imaging probes showed relatively fast blood clearance and low retention in muscle and bone indicating suitable properties for imaging applications matching the short half-life of Gallium-68. The SulfoCy7-conjugate also revealed higher activities in all organs compared to their SulfoCy5 and IRDye800CW counterparts, matching the highest lipophilicity of this conjugate, which was therefore not further explored in imaging studies. Comparable tissue activity was found comparing Sulfo-Cy5 and IRdye800CW conjugates for most organs, except for kidneys where values for IRDye800CW were considerably higher (>20%IA/g). Monomeric MAFC-PEG_5_-Tz conjugates showed comparable biodistribution to non-dye conjugated FSC-based tetrazines [19] and considerably lower activities especially in liver and spleen (~2%IA/g) as compared to FSC-(PEG_5_-Tz)_2_ conjugates with around 10%IA/g. Lung retention was relatively high for all compounds (~5–10%IA/g), no specific reason for this phenomenon could be found.

### 2.5. Imaging Studies

**Figure 5:**
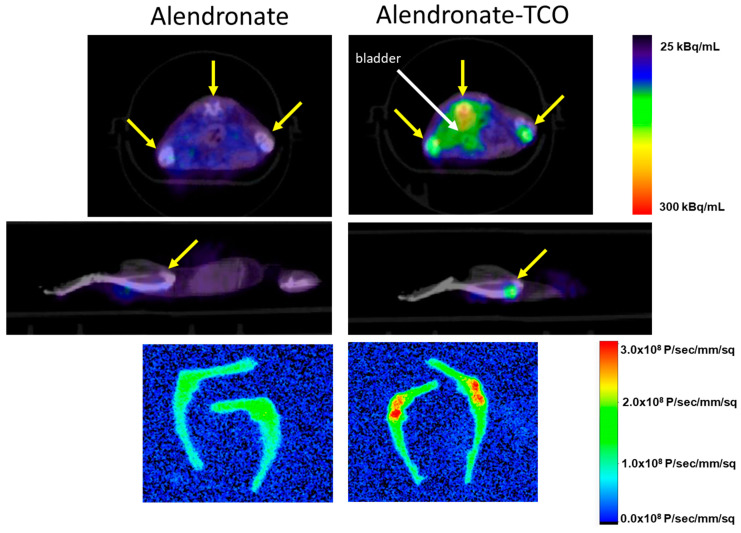
Imaging of ^68^Ga-labeled IRDdye800CW-FSC-(PEG_5_-Tz)_2_ in mice receiving alendronate alone (left row) or alendronate-TCO (right), PET/CT images: transverse slices (top) and sagittal slices (middle), yellow arrows indicate uptake in joints and spine, white arrow indicates the bladder. Bottom image: Optical image of excised bones of the lower limbs. Both PET and OI indicate higher accumulation in bone of alendronate-TCO pre-treated mice as compared to controls.

A simple pretargeting model using bone targeting TCO-alendronate was chosen for a straight forward proof of concept in vivo. PET/CT images revealed specific uptake of both IRDye800CW conjugates in bone structures of TCO-alendronate pre-treated mice, whereas no uptake was seen in mice treated with alendronate alone, proving the in vivo specificity of both constructs. Both conjugates revealed predominant renal excretion and good contrast 1 h p.i. Figure 5 shows images of [^68^Ga]Ga-IRDdye800CW-FSC-(PEG_5_-Tz)_2_ revealing high accumulation in joints and spine with excellent contrast and no uptake in the alendronate control images. This specific accumulation could be confirmed by optical imaging of excised bones with a high accumulation in the joint area, which is not seen in the alendronate control animal. Comparable images of [^68^Ga]Ga- IRDye800CW-MAFC-PEG_5_-Tz are shown in supplementary data (Figure S1) indicating less pronounced uptake in comparison with its monomeric counterpart both in PET/CT and optical images, however without being statistically significant. This is in line with the higher in vitro binding (Figure 2) and our findings when comparing non derivatized mono- and dimeric Tetrazine-FSC conjugates [19], showing that dimerization has a positive effect on in vivo targeting properties. Overall, this proves that dye-conjugated tetrazines based on the FSC scaffold can be used for pretargeting applications combining PET and optical imaging. Further studies, ideally in appropriate pretargeting tumor models, are required to judge the full potential of these compounds for tumor imaging and image-guided procedures.

This is to our knowledge the first report on combining PET and NIRF imaging based on a single tetrazine based pretargeting vector. The FSC scaffold, which is a versatile chelator for Gallium-68, was well suited to develop this approach. In contrast to other attempts, where the dye was conjugated to an antibody [16,17], our approach allows a DMI application without modifying the actual targeting vector, thereby avoiding separate development and characterization of the two versions with and without the dye conjugate.

## 3. Materials and Methods

### 3.1. Analytics

Analytical [radio]-RP-HPLC. Reversed-phase high-performance liquid chromatography analysis was performed with the following instrumentation: UltiMate 3000 RS UHPLC pump, UltiMate 3000 autosampler, Ultimate 3000 column compartment (25 °C oven temperature), UltiMate 3000 Variable Wavelength Detector (Dionex, Germering, Germany; UV detection at λ = 220 nm) a radio detector (GabiStar, Raytest; Straubenhardt, Germany), Jupiter 5 μm C_18_ 300 Å 150 × 4.6 mm (Phenomenex Ltd., Aschaffenburg, Germany) column with acetonitrile (ACN)/H_2_O/0.1% trifluoroacetic acid (TFA) as mobile phase; flow rate of 1 mL/min; gradient: 0.0–1.0 min 10% ACN, 1.0–12.0 min 10–60% ACN, 13.0–15.0 min 60–80% ACN, 15.0–16.0 min 80–10% ACN, and 16.0–20.0 min 10% ACN.

Preparative RP-HPLC. Sample purification via RP-HPLC was carried out as follows: Gilson 322 Pump with a Gilson UV/VIS-155 detector (UV detection at λ = 220 nm) using a PrepFC™ automatic fraction collector (Gilson, Middleton, WI, USA), Eurosil Bioselect Vertex Plus 30 × 8mm 5 μm C_18A_ 300 Å pre-column and Eurosil Bioselect Vertex Plus 300 × 8mm 5 μm C_18A_ 300 Å column (Knauer, Berlin, Germany) and following ACN/H_2_O/0.1% TFA gradients with a flow rate of 2 mL/min: gradient A: 0.0–5.0min 0% ACN, 5.0–35 min 0–50% ACN, 35.0–38.0 min 50% ACN, 38.0–40.0 min 50%–0 ACN. gradient B: 0.0–5.0 min 10% ACN, 5.0–40.0 min 10–60% ACN, 41.0–45.0 min 60% ACN, 46.0–50.0 min 60–80% ACN, and 51.0–55.0 min 80–10% ACN.

#### Mass Spectrometry

Mass analysis was conducted on a Bruker microflex™ bench-top MALDI-TOF MS (Bruker Daltonics, Bremen, Germany) using dried-droplet method on a micro scout target (MSP96 target ground steel BC, Bruker Daltonics) with α-cyano-4-hydroxycinnamic acid (HCCA, Sigma-Aldrich, Handels GmbH, Vienna, Austria) as matrix. Flex Analysis 2.4 software (Bruker Daltonics, Bremen, Germany) was used for data processing.

### 3.2. Synthesis

General. All chemicals and solvents were obtained as reagent grade from commercial sources unless otherwise stated. *Trans*-Cyclooctene-NHS ester and Tetrazine-PEG_5_-NHS ester were purchased from Click Chemistry Tools (Scottsdale, AZ, USA). Rituximab (MabThera^®^, Roche Pharma AG, Grenzach-Wyhlen, Germany) was a kind gift from the University Hospital of Innsbruck. The fluorescent dyes, SulfoCyanine5-NHS ester and SulfoCyanine7 carboxylic acid were obtained from Lumiprobe GmbH (Hannover, Germany) while IRDye800CW carboxylic acid was purchased from LI-COR Biosciences GmbH (Bad Homburg, Germany).

#### 3.2.1. [Fe]fusarinine C ([Fe]FSC) and [Fe]*N*-Monoacetylfusarinine C ([Fe]MAFC)

The macrocyclic iron protected precursors were obtained according to previously published procedures [19]. Briefly, [Fe]FSC was directly isolated from fungal culture in high yield and sufficient chemical purity (>90%, UV–Vis, λ = 220 nm). The monoacetylated derivative [Fe]MAFC could be isolated with reasonable yield and in high purity (>99% UV-detection, λ = 220 nm) via preparative RP-HPLC from a mixture of mono- and multiple acetylated [Fe]FSC-derivatives when reacting [Fe]FSC with acetic anhydride. Both derivatives gave a red−brown colored solid after lyophilization.

- [Fe]FSC: analytical RP-HPLC t_R_ = 6.95 min; MALDI TOF-MS: *m*/*z* [M + H]^+^ = 779.93 [C_33_H_51_FeN_6_O_12_; M*_r_* = 779.63 (calculated)]
- [Fe]MAFC analytical RP-HPLC t_R_ = 7.67 min; MALDI TOF-MS: *m*/*z* [M + H]^+^ = 822.04 [C_35_H_53_FeN_6_O_13_; M*_r_* = 821.67 (calculated)]

#### 3.2.2. Conjugation of PEGylated tetrazine (PEG_5_-Tz)

Either [Fe]FSC (8.0 mg, 10.3 µmol) or [Fe]MAFC (11.0 mg, 13.4 µmol) was dissolved in 500 µL dry DMF and after pH adjustment with DIPEA (pH 9) the mixture was stirred for 30 min at RT. Tetrazine-PEG_5_-NHS (1 equivalent) was dissolved in 200 µL anhydrous DMF and was slowly added dropwise to the solution over a period of 15 min. After 2 h reaction time at ambient temperature the organic solvent was concentrated in vacuo and purified by preparative RP-HPLC using gradient B to give [Fe]FSC-(PEG_5_-Tz)_2_ (t_R_ = 32.9 min) and [Fe]MAFC-PEG_5_-Tz (t_R_ = 26.9 min) as red-brown colored solid after lyophilization. Analytical data:

- [Fe]MAFC-PEG_5_-Tz: 12.5 mg [9.5 µmol, 71%], RP-HPLC t_R_ = 10.2 min; MALDI TOF-MS: *m*/*z* [M + H]^+^ = 1312.21 [C_58_H_84_FeN_11_O_20_; M*_r_* = 1311.19 (calculated)].
- [Fe]FSC-(PEG_5_-Tz)_2_: 4.76 mg [2.71 µmol, 33%], RP-HPLC t_R_ = 11.4 min; MALDI TOF-MS: *m*/*z* [M + H]^+^ = 1759.03 [C_79_H_113_FeN_16_O_26_; M*_r_* = 1758.68 (calculated)].

#### 3.2.3. Synthesis of Monomeric FSC-based Tz Hybrid Imaging Agents

For conjugation of the fluorescent dyes to the monomeric FSC-based Tz-ligand, 2.3 mg of [Fe]MAFC-PEG_5_-Tz (1.75 µmol) were each dissolved in 500 µL dry DMF, pH was adjusted with DIPEA (pH 9) and 1.1 equivalent of the corresponding dye dissolved in 500 µL DMF was added. SulfoCyanine5-NHS ester (1.50 mg, 1.93 µmol) was added directly while the carboxylic acid of SulfoCyanine7 (1.41 mg, 1.93 µmol), as well as IRDye800CW (2.1 mg, 1.93 µmol) were pre-activated with 1.5 equivalent of *O*-(7-Azabenzotriazol-1-yl)-*N*,*N*,*N′*,*N′*-tetramethyluronium-hexafluorphosphate (HATU, 1.1 mg, 2.89 µmol) for 10 min at ambient temperature. The reaction mixtures were maintained for complete conjugation at RT for 6 h followed by evaporation of DMF in vacuo. The crude bioconjugates were re-dissolved in 1 mL 50% ACN/H_2_O (*v*/*v*). Half of the solution (500 µL) was purified by preparative RP-HPLC (gradient B) to obtain SulfoCyanine5-[Fe]MAFC-Tz (t_R_ = 32.0 min) as dark blue and SulfoCyanine7-[Fe]MAFC-Tz (t_R_ = 34.9 min) as well as IRDye800CW-[Fe]MAFC-Tz (t_R_ = 28.8 min) as dark green colored solids after lyophilization. Analytical data:

- SulfoCyanine5-[Fe]MAFC-PEG_5_-Tz: 0.61 mg [0.32 µmol, 36%], RP-HPLC t_R_ = 10.6 min; MALDI TOF-MS: *m*/*z* [M + H]^+^ = 1936.99 [C_90_H_120_FeN_13_O_27_S_2_; M*_r_* = 1935.96 (calculated)]
- SulfoCyanine7-[Fe]MAFC-PEG_5_-Tz: 0.85 mg [0.42 µmol, 49%], RP-HPLC t_R_ = 11.2 min; MALDI TOF-MS: *m*/*z* [M + H]^+^ = 2002.85 [C_95_H_126_FeN_13_O_27_S_2_; M*_r_* = 2002.06 (calculated)]
- IRDye800CW-[Fe]MAFC-PEG_5_-Tz: 1.11 mg [0.48 µmol, 55%], RP-HPLC t_R_ = 9.5 min; MALDI TOF-MS: *m*/*z* [M + H]^+^ = 2297.02 [C_104_H_136_FeN_13_O_34_S_4_; M*_r_* = 2296.36 (calculated)]

The remaining half of the reaction mixture (500 µL) was used for demetallation. Therefore, 1 mL of disodium EDTA (Na_2_EDTA, 200 mM) was added and the mixture was stirred for 4 h at RT to completely remove the iron from the conjugates followed by preparative RP-HPLC purification to give intensively green to blue colored solids after freeze drying. Analytical data:

- SulfoCyanine5-MAFC-PEG_5_-Tz: 0.55 mg [0.29 µmol, 34%], gradient B (t_R_ = 32.5 min); Analytical data: RP-HPLC t_R_ = 10.8 min; MALDI TOF-MS: *m*/*z* [M + H]^+^ = 1883.75 [C_90_H_123_N_13_O_27_S_2_; M*_r_* = 1883.14 (calculated)]
- SulfoCyanine7-MAFC-PEG_5_-Tz: 0.70 mg [0.36 µmol, 41%], gradient B (t_R_ = 35.5 min); Analytical data: RP-HPLC t_R_ = 11.4 min; MALDI TOF-MS: *m*/*z* [M + H]^+^ = 1949.70 [C_95_H_129_N_13_O_27_S_2_; M*_r_* = 1949.24 (calculated)]
- IRDye800CW-MAFC-PEG_5_-Tz: 1.23 mg [0.55 µmol, 63%], gradient B (t_R_ = 29.2 min); Analytical data: RP-HPLC t_R_ = 9.7 min; MALDI TOF-MS: *m*/*z* [M + H]^+^ = 2244.26 [C_104_H_139_N_13_O_34_S_4_; M*_r_* = 2243.54 (calculated)]

#### 3.2.4. Synthesis of Dimeric FSC-based Tz Hybrid Imaging Agents

Conjugation of the fluorescent dye to the dimeric FSC-based Tz-ligand was performed as described above using 1.0 mg of [Fe]FSC-(PEG_5_-Tz)_2_ (0.57 µmol) as starting material. After successful conjugation, demetallation was performed as described above followed by purification via preparative RP-HPLC. Analytical data:

- SulfoCyanine5-FSC-(PEG_5_-Tz)_2_: 0.80 mg [0.34 µmol, 60%], gradient B (t_R_ = 36.7 min); Analytical data: RP-HPLC t_R_ = 11.61 min; MALDI TOF-MS: *m/z* [M + H]^+^ = 2332.75 [C_111_H_153_N_18_O_33_S_2_; M*_r_* = 2331.63 (calculated)]
- IRDye800CW-FSC-(PEG_5_-Tz)_2_: 0.61 mg [0.22 µmol, 40%], gradient B (t_R_ = 31.5 min); Analytical data: RP-HPLC t_R_ = 11.10 min; MALDI TOF-MS: m/z [M + H]^+^ = 2693.40 [C_125_H_169_N_18_O_40_S_4_; M*_r_* = 2692.03 (calculated)]

#### 3.2.5. Modification of Rituximab (RTX)

Anti-CD20 monoclonal antibody Rituximab was modified as previously reported [19]. Briefly, the antibody solution (Mabthera^®^, 10 mg/mL, Roche Pharma AG, Grenzach-Wyhlen, Germany) was allowed to react at 4 °C overnight in 0.1 M NaHCO_3_ solution with 20 molar equivalent of TCO–NHS ester dissolved in DMSO followed by size exclusion column purification via PD-10 (GE Healthcare Vienna, Austria) to give RTX–TCO in PBS (2.5 mg/mL).

### 3.3. Radiochemistry

Radiolabelling with Gallium-68 was performed by mixing 500 µL [^68^Ga]gallium chloride ([^68^Ga]GaCl_3_) eluate, obtained by fractioned elution of a ^68^Ge/^68^Ga-generator (IGG100, nominal activity 1850 MBq, Eckert and Ziegler, Berlin, Germany) with 0.1 M hydrochloric acid (HCl, Rotem Industries Ltd., Beer-Sheva, Israel), with 100 µL sodium acetate solution (1.14 M) to give a final pH of 4.5 and adding 10 µg (3.71-5.31 nmol) of the corresponding fluorescent FSC-Tz derivative. After incubation for 5 min at ambient temperature radio-RP-HPLC- as well as radio-ITLC-analysis was performed.

#### Radio-ITLC

Instant thin layer chromatography (ITLC) analysis was performed using TLC-SG strips (Varian, Lake Forest, CA, USA) as stationary phase and 0.1 M sodium citrate solution (pH 5) or 1 M ammonium acetate/methanol (1:1, *v/v*) with a pH of 6.8 as mobile phase. The strips were analyzed using a TLC scanner (Scan-RAM^™^, LabLogic, Sheffield, UK).

### 3.4. In Vitro Characterization

#### 3.4.1. Distribution Coefficient (logD)

To determine the hydrophilicity the distribution of the ^68^Ga-labeled conjugates between an organic (octanol) and aqueous (PBS) layer was assessed. Aliquots (50 µL) of the ^68^Ga-labeled tracers (~5 µM) were diluted in 1 mL of octanol/PBS (1:1, *v/v*) and the mixture was vortexed at 1400 rpm (MS 3 basic vortexer, IKA, Staufen, Germany) for 15 min at RT. Subsequently the mixture was centrifuged for 2 min at 4500 rpm followed by measuring aliquots (50 µL) of both layers in the gamma counter (Wizard^2^ 3”, Perkin Elmer, Waltham, MA, USA). LogD was calculated using Microsoft Excel (*n =* 3, six replicates).

#### 3.4.2. Protein Binding

The ability of the ^68^Ga-labeled conjugates to bind to serum proteins was determined by size exclusion chromatography. Aliquots (50 µL, *n =* 3) of the radioligand solution (~10 µM) were incubated in 450 µL freshly prepared human serum as well as 450 µL PBS as control and the mixtures were maintained at 37 °C. After 1, 2, and 4 h, aliquots (25 µL) were transferred to illustra MicroSpin G-50 columns (GE Healthcare Vienna, Austria) and the percentage of protein-bound (eluate) and non-bound (column) conjugate was calculated.

#### 3.4.3. Cell-Binding Studies

CD20-expressing Raji cells (humanoid lymphoblast-like B-lymphocyte cells) were obtained from the American Type Culture Collection (ATCC, Manassas, Virginia, USA). The cells were cultured in tissue culture flasks (Cellstar; Greiner Bio-One, Kremsmuenster, Austria) using RPMI-1640 medium with 10% (*v/v*) fetal bovine serum (FBS) as supplement (Invitrogen Corporation, Lofer, Austria). For cell binding studies 10 × 10^6^ cells were washed twice with fresh medium, diluted with PBS to a final concentration of 1 × 10^6^ cells per mL and 500 µL of cell suspension was transferred to Eppendorf tubes. Hereafter, 50 µL of RTX–TCO or non-modified RTX as negative control (both 0.5 µM) was added and the cell suspension was maintained at 37 °C under gentle shaking. After 1 h the suspension was centrifuged (2 min, 11 × 10^3^ rcf), the supernatant was discarded and the cells were washed twice with 600 µL PBS and finally resuspended with 450 µL PBS. Subsequently 50 µL of the radioligand solution (22 nM) was added and the suspension was incubated for 30 min at 37 °C. After centrifugation and two washing steps with 600 µL PBS, the cells were resuspended in 500 µL PBS and transferred to polypropylene vials for gamma counter measurement followed by calculation of cell-associated activity in comparison to the total activity applied (*n =* 3, six replicates).

#### 3.4.4. Fluorescence Microscopy

CD20-expressing Raji cells were prepared and pre-treated with modified or non-modified RTX as described above (cell-binding studies). Instead of the radioligand, 50 µL (22 nM) of iron protected SulfoCyanine5-[Fe]MAFC-PEG_5_-Tz was added and the cells were treated according to the cell-binding studies with the ^68^Ga-labeled counterparts. After resuspension fluorescence microscopy was performed using a laser (λ = 561 nm) for excitation of the fluorescent dye. The imaging was carried out with a spinning-disc confocal microscopic system (Ultra VIEW VoX, PerkinElmer, Waltham, MA, USA) linked to a Zeiss AxioObserver Z1 inverted microscope (Zeiss, Oberkochen, Germany). Images were acquired with Volocity software (PerkinElmer) utilizing a 63× oil immersion objective (numerical aperture 1.42). The images show z-stacks (*n* = 4; 1 µm spacing). Cell morphology was visualized by adding fluorescently labeled wheat germ agglutinin (Alexa Fluor^®^ 488 WGA, ThermoFischer Scientific, Vienna, Austria).

### 3.5. In Vivo Characterization

Ethics statement: All animal experiments were performed in accordance with regulations and guidelines of the Austrian animal protection laws and the Czech Animal Protection Act (No. 246/1992), with approval of the Austrian Ministry of Science (BMWF-66.011/0161-WF/V/3b/2016) and the Czech Ministry of Education, Youth, and Sports (MSMT-18724/2016-2), and the institutional Animal Welfare Committee of the Faculty of Medicine and Dentistry of Palacky University in Olomouc, Czech Republic.

#### 3.5.1. Biodistribution Studies

Biodistribution studies of the FSC-based hybrid imaging agents radiolabeled with Gallium-68 were performed using healthy five-week-old female BALB/c mice (Charles River Laboratories, Sulzfeld, Germany). Animals (*n =* 3) were injected via lateral tail vein with 1 nmol of conjugate and a total activity of approximately 6 MBq. Mice were sacrificed by cervical dislocation 1 h p.i. followed by collection of the main organs and tissue, subsequent gamma counter measurement and calculation of the percentage of injected activity per gram tissue (% IA/g).

#### 3.5.2. Imaging Studies

##### Pretargeting Model

For the proof of concept of pretargeting in vivo a model described by Yazdani et al. was applied [20], based on TCO-modified alendronate for bone targeting. For preparation of TCO–alendronate, briefly, 20 mg sodium alendronate trihydrate (Sigma Aldrich) was dissolved in 400 µL 0.1N sodium-bicarbonate solution pH 8.5. Then 0.6 mL TCO–NHS ester (Click chemistry tools, Scottsdale, AZ, USA), 20 mg/mL in DMSO, were slowly added to the solution and stirred overnight in the dark, followed by freeze drying. As a control the same preparation was prepared, replacing TCO–NHS solution by the same volume of DMSO. For injection in mice the residue was dissolved in water and adjusted to pH 7 (30 mM).

##### PET/CT Imaging

MicroPET/CT images were acquired with an Albira PET/SPECT/CT small animal imaging system (Bruker Biospin Corporation, Woodbridge, CT, USA). Healthy 10-week-old female BALB/c mice (Envigo, Horst, The Netherlands) (*n* = 4) were pre-treated by intraperitoneal injection of 250 µL corresponding to 4 mg of alendronate-TCO or 2.5 mg alendronate alone, respectively. Pre-treated mice were retro-orbitally injected with radiolabeled tracer in a dose of 5–10 MBq corresponding to 1–2 μg of conjugate per animal 5 h after the pre-treatment. Anaesthetized (2% isoflurane (FORANE, Abbott Laboratories, Abbott Park, IL, USA)) animals were placed in a prone position in the Albira system before the start of imaging. Static PET/CT images were acquired over 30 min starting 1 h p.i.. A 10-min PET scan (axial FOV 148 mm) was performed, followed by a double CT scan (axial FOV 2 × 65 mm, 45 kVp, 400 μA, at 400 projections). Scans were reconstructed with the Albira software (Bruker Biospin Corporation, Woodbridge, CT, USA) using the maximum likelihood expectation maximization (MLEM) and filtered back projection (FBP) algorithms. After reconstruction, acquired data was viewed and analyzed with PMOD software (PMOD Technologies Ltd., Zurich, Switzerland).

##### Optical Imaging

Near-infrared in vivo fluorescence imaging was performed with an In-Vivo MS FX PRO small animal imaging system (Bruker Biospin Corporation, Woodbridge, CT, USA) after the PET/CT investigation. Mice injected with the different conjugates were sacrificed 24 h p.i. and excised bones of the lower limbs were imaged. An appropriate filter set (λ_ex_ = 710 nm and λ_em_ = 790 nm) was used for acquiring the fluorescence of the IRDye800CW-conjugates ex vivo. Identical illumination settings (acquisition time = 30 s, filters = 710/790 nm, f-stop = 2.8, field of view = 100 mm, and binning = 2 × 2) were used for image acquisition and fluorescence emission was normalized to photons/s/mm^2^. Acquired images were analyzed using Bruker MI SE software (Bruker Biospin Corporation, Woodbridge, CT, USA).

### 3.6. Statistical Analysis

Statistical analysis was performed using independent two-tailed Student’s T-Test with *P*-value < 0.05 indicating significance.

## 4. Conclusions

We synthesized a series of tetrazines combining radiolabeling for PET with optical NIR imaging on a single scaffold. A Sulfo-Cy7 conjugate was shown to have suboptimal properties but versions based on Sulfo-Cy5 and IRDye800CW revealed specific binding to TCO-modified targets in vitro and specific accumulation in vivo exemplified by imaging. A dimeric version showed enhanced and more specific signal in vivo, making this a promising candidate to further investigate multimodality imaging techniques based on pretargeting strategies.

## Figures and Tables

**Table 1:** Distribution coefficient (logD) and protein binding of ^68^Ga-labeled FSC-based Tz-bearing hybrid imaging agents.

^68^Ga-Labeled Conjugate	Distribution Coefficient	Protein Binding (%)		
	logD (pH 7.4)	1 h	2 h	4 h
SulfoCy5-MAFC-PEG_5_-Tz	−2.85 ± 0.08	37.0 ± 0.3	37.4 ± 2.3	37.6 ± 1.2
SulfoCy7-MAFC-PEG_5_-Tz	−1.92 ± 0.05	36.9 ± 0.8	40.4 ± 1.5	41.6 ± 2.7
IRDye800CW-MAFC-PEG_5_-Tz	−2.40 ± 0.05	65.7 ± 1.3	67.3 ± 1.4	67.7 ± 0.2
SulfoCy5-FSC-(PEG_5_-Tz)_2_	−2.29 ± 0.10	35.7 ± 0.7	39.1 ± 0.4	40.8 ± 0.6
IRDye800CW-FSC-(PEG_5_-Tz)_2_	−2.46 ± 0.09	47.3 ± 0.2	50.3 ± 0.3	54.9 ± 1.1

Data are presented as mean ± SD (*n* = 3).

## Supplementary Materials

The following are available online, Figure S1: OI and PET Imaging of ^68^Ga-labeled IRDdye800CW-MAFC-PEG_5_-Tz in mice.
