# Supplementary material for: Hybrid Imaging Agents for Pretargeting Applications Based on Fusarinine C—Proof of Concept
Source: Molecules. 2020 May 1;25(9):2123. doi: 10.3390/molecules25092123 (PMC7249120; doi:10.3390/molecules25092123)

Supplementary data

Figure S1 Imaging of ^{68}Ga-labelled IRDdye800CW-MAFC-PEG5-Tz in mice receiving Aledronate alone (left row) or Aledronate-TCO (right), PET-CT images: transvers slices (top) and sagittal slices (middle), yellow arrows indicate uptake in joints and spine, white arrow indicates the bladder. Lower image: Optical image of exczised bone of lower limbs. BOTH PET and OI indicate higher accumulation in bone of Alendronate-TCO pre-treated mice as compared to controls

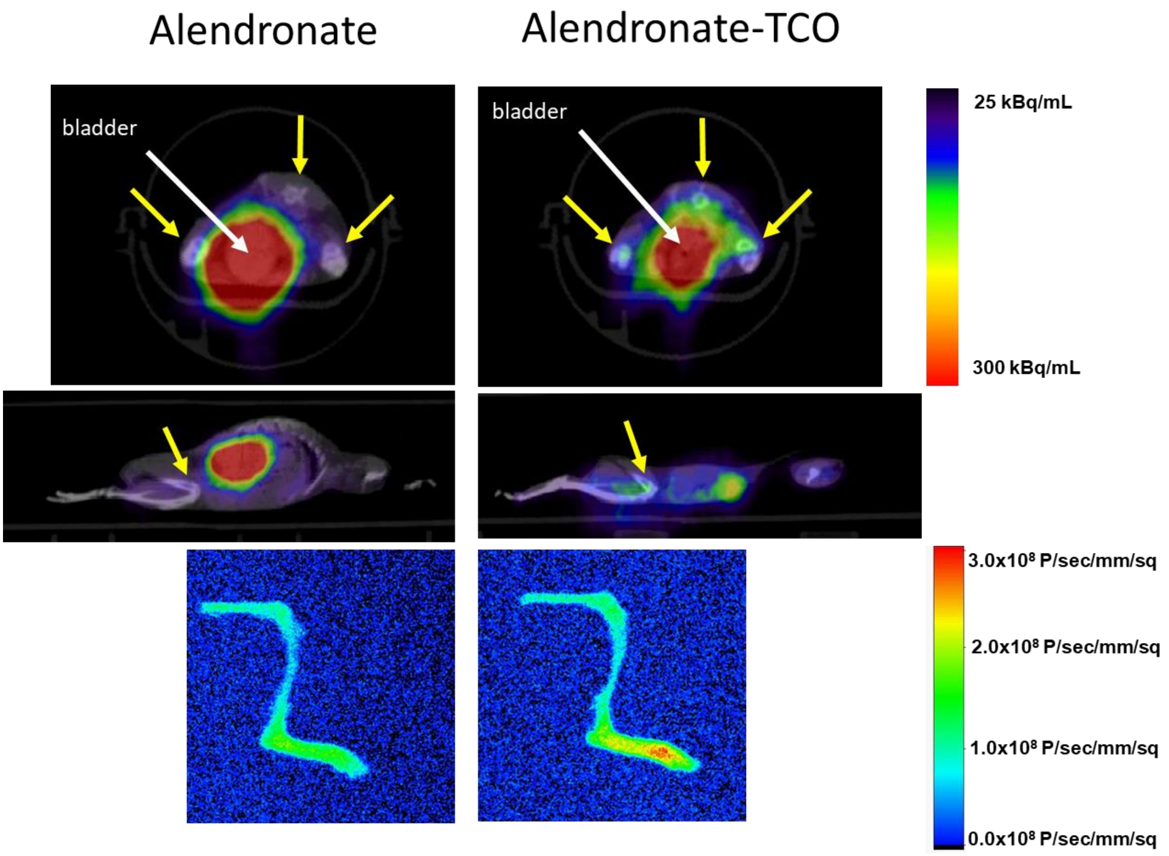

Supplement: Supplementary file 1 [file molecules-25-02123-s001.pdf]
